# Busting the Resistance: Antimicrobial Activity of Plant-Infused Nanoemulsions against *Neisseria gonorrhoeae*

**DOI:** 10.1155/2024/7084347

**Published:** 2024-07-30

**Authors:** Deshanta Naicker, Rowen Govender, Nathlee S. Abbai

**Affiliations:** School of Clinical Medicine Laboratory College of Health Sciences Nelson R. Mandela School of Medicine University of KwaZulu-Natal, Durban, South Africa

## Abstract

The escalating antibiotic resistance rates in *Neisseria gonorrhoeae* (*N. gonorrhoeae*) are now a grave concern. There is a critical need for alternative treatment options for infection since *N. gonorrhoeae* has developed resistance to multiple antibiotics used for treatment. In this study, plant nanoemulsions from *Ocimum tenuiflorum*, *Moringa oleifera*, and *Azadirachta indica* were tested for their antimicrobial properties against *N. gonorrhoeae.* The study also assessed the toxicity of these plant nanoemulsions using human erythrocytes. The plants were sourced from the Botanical Gardens in Durban, South Africa. Nanoemulsions were produced from the dried plants using established methods. The nanoemulsion-based plant extracts were tested against laboratory (World Health Organization (WHO) strains) and clinical isolates of *N. gonorrhoeae* using the disk diffusion method. All six isolates had zones of inhibition for the 1000 *μ*M concentration for all three nanoemulsion-based plant extracts. No zones of inhibition were observed for 100 *μ*M, 10 *μ*M, and 1 *μ*M nanoemulsion concentrations for five of the isolates. Isolate G176 had zones of inhibition at 1000 *μ*M and 100 *μ*M concentrations for the nanoemulsions of *Ocimum tenuiflorum*. Both the WHO strains had zones of inhibition appearing at the 1000 *μ*M concentration. For the WHO Y strain, zones of inhibition for both 1000 *μ*M and 100 *μ*M concentrations were observed for the nanoemulsions of *Ocimum tenuiflorum* and *Azadirachta indica*. According to the analysis, there was 0% haemolytic activity observed which suggests the nontoxic nature of the extracts. This study showed that the nanoemulsion and plant mix may potentially be used as a safer alternative to treat gonorrhoea.

## 1. Introduction

Sexually transmitted infections (STIs) continue to be a global public health concern, with *Neisseria gonorrhoeae* (*N. gonorrhoeae*), the Gram-negative bacterium responsible for gonorrhoea, posing challenges due to its ability to develop antibiotic resistance [1–5]. South Africa has followed the recommendations made by the WHO in 2014, which advocated for the replacement of the first-line treatment with oral cefixime to a single injectable dose (250 mg) of ceftriaxone [4, 6]. Treatment failures of ceftriaxone monotherapy led to the WHO recommendation of administering dual antimicrobial therapy with the combination of ceftriaxone (250 mg) and azithromycin (1 g stat) [4]. However, decreasing susceptibility of *N. gonorrhoeae* to ceftriaxone has been reported with the proportion of resistance to ceftriaxone varying extensively, from 1.3% to 55.8% [7]. In addition, resistance to azithromycin is already prevalent in many settings [8]. Therefore, dual antimicrobial therapy cannot ensure long-term effectiveness. This highlights the critical need to develop an alternative therapeutic to reduce the spread of *N. gonorrhoeae*. The emergence of multidrug-resistant strains of *N. gonorrhoeae* has heightened the urgency for innovative and effective treatment strategies. In this study, the fusion of two promising fields of research, nanoemulsion technology and plant-based therapeutics, presents a novel approach to combating *N. gonorrhoeae* infections.

Nanoemulsions, which are currently the subject of extensive investigation, serve as submicron-sized emulsions utilized for the improved delivery of therapeutic agents. They are recognized as an advanced nanoparticle technique that contributes to the more efficient systemic administration of biologically active substances, ultimately enhancing and regulating drug delivery [9]. A nanoemulsion is defined as a stable, thermodynamically balanced, clear dispersion consisting of two immiscible liquids, typically oil and water. This stability is achieved through the presence of an interfacial layer formed by surfactant molecules [9–11]. The dispersed phase generally consists of minuscule particles or droplets, falling within the size range of 5 nm–200 nm, and possesses an extremely low interfacial tension between the oil and water phases. Its formation is facilitated because the droplet size is less than 25% of the wavelength [9, 10]. In many instances, a co-surfactant or co-solvent is included, along with the surfactant, oil phase, and water phase, resulting in three essential components within a nanoemulsion [9–13]. Nanotechnology has greatly augmented the potential for drug targeting, reducing the necessary dosage size and quantity for effective treatment [13]. Consequently, the study's objective is to explore the utilization of nanotechnology in conjunction with plant extracts to identify an alternative therapeutic approach for combating *N. gonorrhoeae* infections. By combining the unique properties of nanoemulsions, such as enhanced drug delivery and stability, with the diverse bioactive compounds found in plant extracts, we hope to unlock new solutions in the battle against this persistent and adaptable bacterial pathogen [13].

The properties found in plant extracts have been harnessed in recent times to counteract the adverse effects of pharmaceutical drugs and mitigate the rise of pathogens that are resistant to multiple drugs [10]. This study will investigate the antimicrobial properties of *Ocimum tenuiflorum* (“holy basil”), *Moringa oleifera* (*M. oleifera*), and *Azadirachta indica* (*A. indica*) plants. These plants are suggested to have antibacterial and anti-inflammatory properties and may show promising effects against *N. gonorrhoeae* [14–18]. While these plants, *Ocimum tenuiflorum* (*O. tenuiflorum*), *M. oleifera*, and *A. indica* have been traditionally used for their potential medicinal properties, their antimicrobial properties against STIs require more comprehensive scientific research. Therefore, this will be the first study to explore the activity of these plants against *N. gonorrhoeae* in South Africa.

This study aimed to determine the antimicrobial activities of plant nanoemulsions from *O. tenuiflorum*, *M. oleifera*, and *A. indica* plants against *N. gonorrhoeae* and to determine the toxicity of the plant nanoemulsions using human erythrocytes.

## 2. Materials and Methods

### 2.1. Ethical Approval

Ethical approval for this study was obtained from the Biomedical Research Ethics Committee (BREC) of the University of KwaZulu-Natal, (BREC/00005104/2022).

### 2.2. Study Design

This study was a retrospective laboratory-based study using stored clinical isolates. For this analysis, six clinical isolates testing positive for *N. gonorrhoeae*, along with two WHO reference strains (WHO Y and WHO Z), were utilized. The clinical isolates were obtained from endocervical swabs collected from six pregnant women at King Edward VIII Hospital in Durban, South Africa, between October 2018 and April 2021. A clinician performed the collection of the endocervical swabs. The WHO strains were generously provided by the National Institute of Communicable Diseases (NICD), South Africa. The WHO Y and WHO Z isolates were both ceftriaxone and azithromycin resistant [19]. Resistance for ceftriaxone and azithromycin was tested against the six clinical isolates using Etest™ method (BioMérieux, France); however, all were susceptible.

### 2.3. Culture Detection of *N. gonorrhoeae*

The stored isolates were plated out onto chocolate agar plates (ThermoFisher Scientific, United States) and incubated for 24 to 48 hours at 37°C in the presence of 5% CO_2_. After this incubation process, suspected colonies were subcultured onto chocolate agar plates using a four-way steak. The plates were incubated for another 24 to 48 hours in the presence of 5% CO_2_. Presumptive tests were conducted which included the oxidase test, Gram-staining, and the API test kit (BioMérieux, United States of America) which acted as a means of confirmation [20].

### 2.4. Plant Collection and Preparation of Extracts

**Figure 1:**
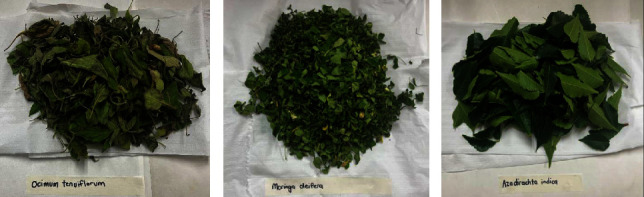
Images of the cleaned and dried leaves of (a) *O. tenuiflorum*, (b) *M. oleifera*, and (c) *A. indica* plants.

The plant leaves (*O. tenuiflorum*, *M. oleifera*, and *A. indica*) were collected from the Botanical Gardens in Durban, South Africa. After removing any dust and dirt, the leaves underwent an initial washing step by immersing them in deionized water for one minute. Subsequently, the leaves were left to naturally air dry for 4-5 days, shielded from direct sunlight for the preparation of aqueous extracts (Figure 1).

**Figure 2:**
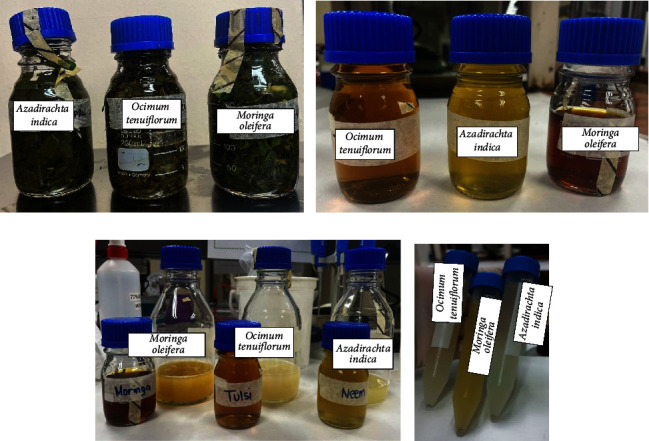
Preparation of the nanoemulsions of the three plants: (a) Image showing the leaves after they had been boiled and cooled down during the plant preparation step. (b) Image showing the leaves after the S1 preparation step. (c) Image showing the nanoemulsion after the S2 preparation step. (d) Image showing the final nanoemulsion with the leave extracts that can be stored at 4°C.

For the preparation of the plant extracts, the dried leaves were cut into small pieces using sterilized scissors and approximately 100 g of each type of leaf material was combined with 500 ml of distilled water and boiled for 30 minutes at 100°C (Figure 2(a)). Following boiling, the mixture was allowed to cool and then filtered to obtain the aqueous extract. The extracts were further filtered using punched Whatman filter paper (no. 1) (Sigma-Aldrich, Germany) and subjected to centrifugation (1500 rpm for 10 minutes) to remove any remaining solid particles and were stored at −20°C until ready for use.

### 2.5. Nanoemulsion of the Plants

Preparation of the oil phase involved creating a homogeneous organic solution (referred to as S1) comprising 400 *μ*l of isopropyl myristate (Sigma-Aldrich, Germany) and 86 *μ*l of span 80 (Sigma-Aldrich, Germany), which is a lipophilic surfactant, dissolved in a water-miscible solvent (Figure 2(b)).

To prepare the aqueous phase, a homogeneous solution (S2) was formed by mixing 80 ml of water with 136 *μ*l of Tween 80 (Sigma-Aldrich, Germany), a hydrophilic surfactant. Into this aqueous phase, 30 ml of the plant extract was introduced while subjecting the mixture to magnetic stirring. This resulted in the nearly instant formation of an oil-in-water (o/w) emulsion as the organic solvent diffused into the external aqueous phase, creating nano-sized droplets. Magnetic stirring was continued for 30 minutes to allow the system to reach equilibrium (Figure 2(c)).

To remove all the water-miscible solvent, the emulsion was subjected to evaporation for 45 minutes under reduced pressure while being centrifuged at 1000 rpm. Subsequently, the emulsion was cooled by immersing it in an ice bath for 10 minutes (Figure 2(d)). Various nanoemulsion concentrations, namely, 1000 *μ*M, 100 *μ*M, 10 *μ*M, and 1 *μ*M, were prepared as part of the process and stored at 4°C.

### 2.6. Antimicrobial Activity of the Nanoemlusions

A disk diffusion method was adapted for this experiment. Punched Whatman filter paper (no. 1) was baked at 160℃ for 1 hour to sterilize the paper and thereafter cooled until ready for use at room temperature. Overnight cultures (Brain Heart Infusion (BHI) medium was used) of the isolates were used to make a 0.5 McFarland using deionized water. Thereafter, 100 *μ*l of the McFarland solution was plated out on chocolate agar plates. The sterile disks were saturated with the nanoemulsions: 5 *μ*l of different concentrations (1000 *μ*M, 100 *μ*M, 10 *μ*M, and 1 *μ*M) of the nanoemulsions were added to the disks aseptically. The disks were then placed on the chocolate agar plate using aseptic techniques. The agar plates were incubated for 24 hours in a 37°C CO_2_ incubator. Antimicrobial activity was recorded as zones of clearance around the disks.

### 2.7. Haemolytic Activity of the Nanoemulsions

Preparation of the erythrocyte suspension employed in this study was similar to the methods reported by Kumar et al. 2011 [21]. Blood was collected from a healthy individual by venepuncture. The blood was immediately processed by centrifugation at 1500 rpm for 10 minutes. The supernatant (plasma) was discarded, and the pellet which contained the erythrocytes was washed three times with sterile phosphate buffer saline (PBS) (Sigma-Aldrich, Germany) solution (pH 7.2 ± 0.2) and centrifuged at 1500 rpm for 5 minutes. A 5% cell suspension was then made using normal saline.

*In vitro*, haemolytic activity was measured by spectrophotometry as described by Yang et al. 2005 [22]. A volume of 100 *μ*l of the cell suspension was mixed with 100 *μ*l of the individual plant extracts. The mixtures were incubated for 30 minutes at 37°C. Following incubation, the mixture was centrifuged at 1500 rpm for 10 minutes. The free haemoglobin in the supernatant was measured in a UV spectrophotometer at 540 nm. The haemolytic controls for this experiment were a PBS control (100 *μ*l of cell suspension and 100 *μ*l PBS) and water control (100 *μ*l cell suspension and 100 *μ*l sterile water). Each experiment was performed in triplicate at each concentration across the individual plant types.

Percentage haemolysis was calculated according to the following formula:

(1) $$\begin{array}{c} \%\text{ Haemolysis}=\frac{A_{T\;}-A_{N}}{A_{C}-A_{N}}\times 100, \end{array}$$

*A*_*T* _ is the absorbance of test sample. *A*_*N*_ is absorbance of the control (saline control). *A*_*C*_ is the absorbance of the control (water control).

## 3. Results

### 3.1. Antimicrobial Activity of the Nanoemulsions

**Figure 3:**
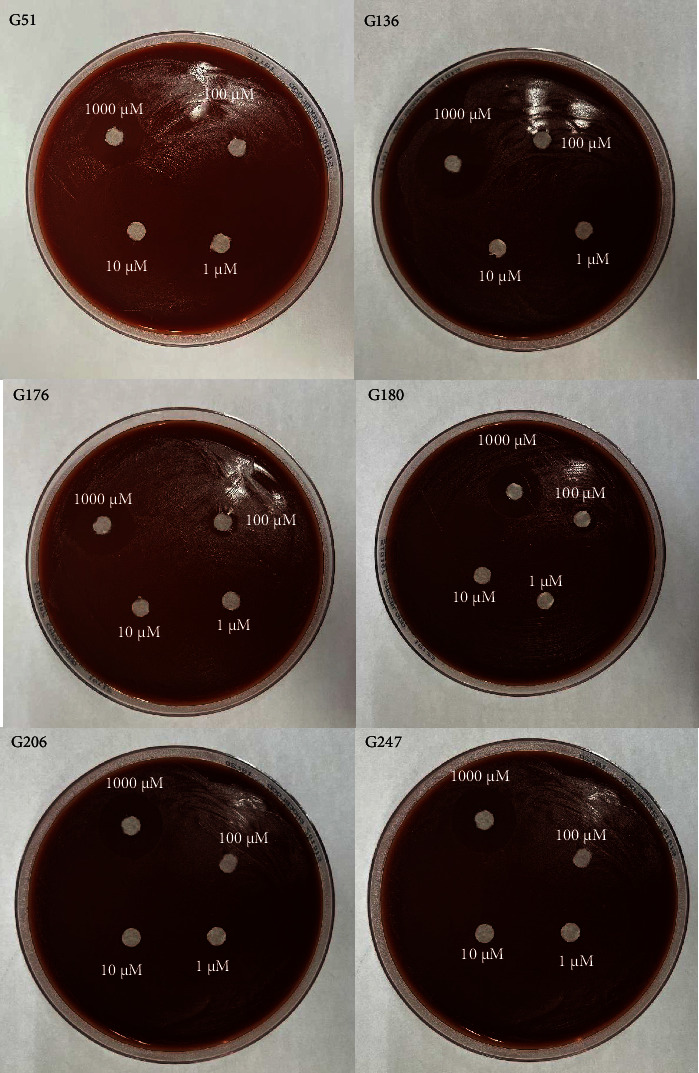
Antimicrobial activity of the nanoemulsions (different concentrations) of *O. tenuiflorum*. At a concentration of 1000 *μ*M of the nanoemulsion, antimicrobial activity against the isolates of *N. gonorrhoeae* was observed. However, for isolate G176, a small zone of inhibition was observed at 100 *μ*M as well.

**Figure 4:**
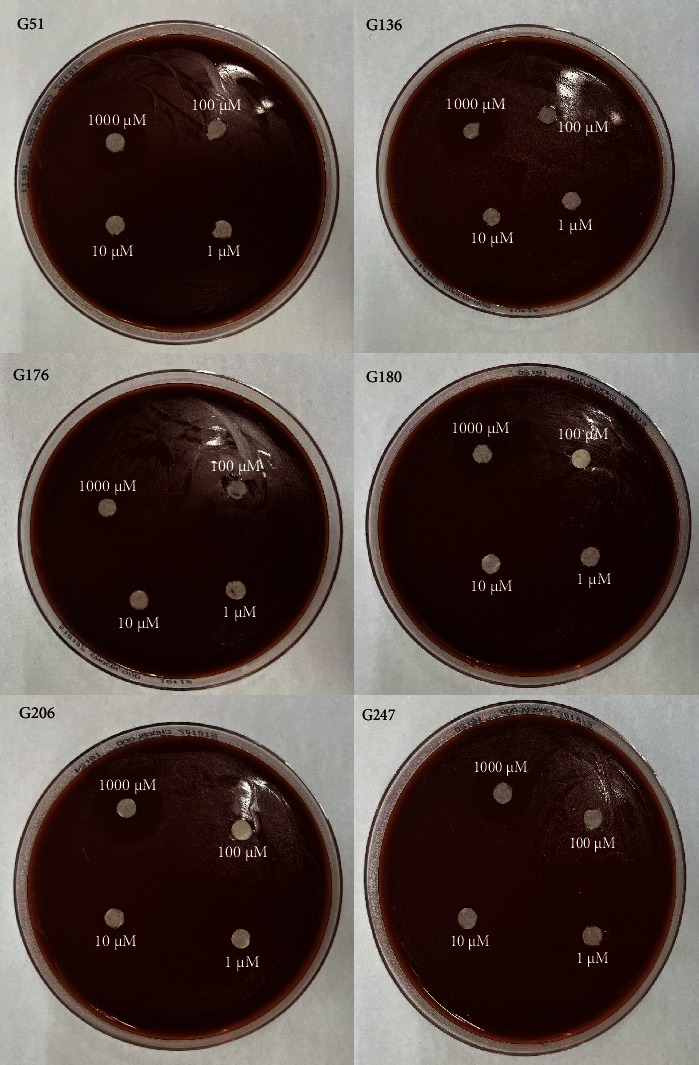
Antimicrobial activity of the nanoemulsions (different concentrations) of *M. oleifera*. At a concentration of 1000 *μ*M of the nanoemulsion, antimicrobial activity against the isolates of *N. gonorrhoeae* was observed.

**Figure 5:**
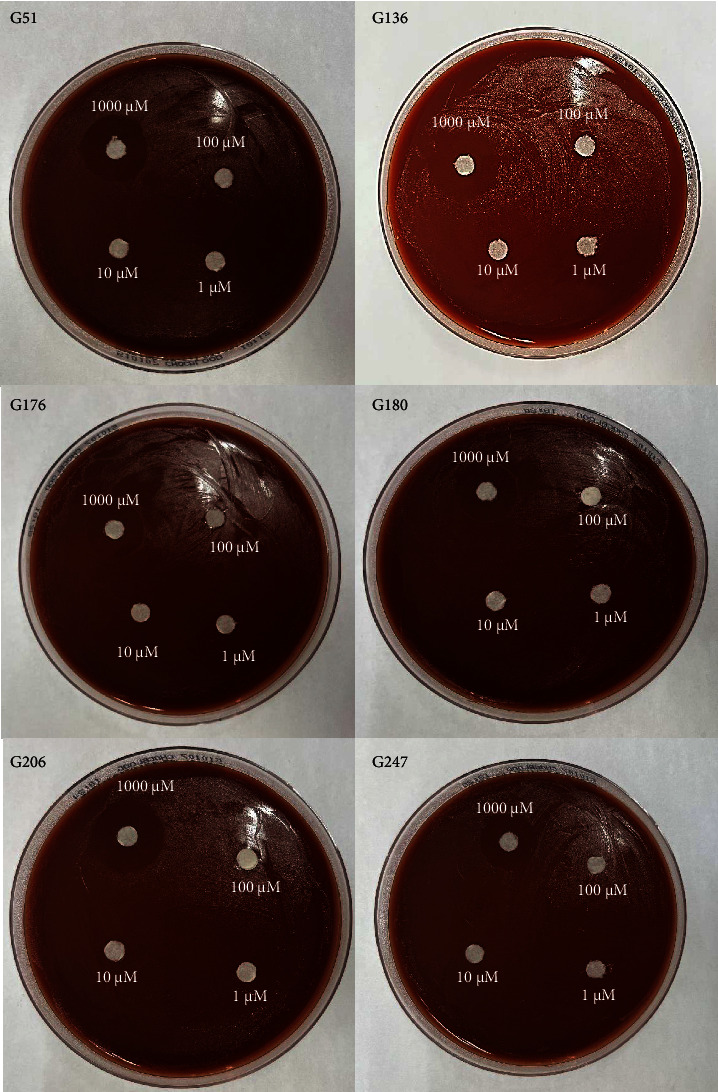
Antimicrobial activity of the nanoemulsions (different concentrations) of *A. indica*. At a concentration of 1000 *μ*M of the nanoemulsion, antimicrobial activity against the isolates of *N. gonorrhoeae* was observed.

**Figure 6:**
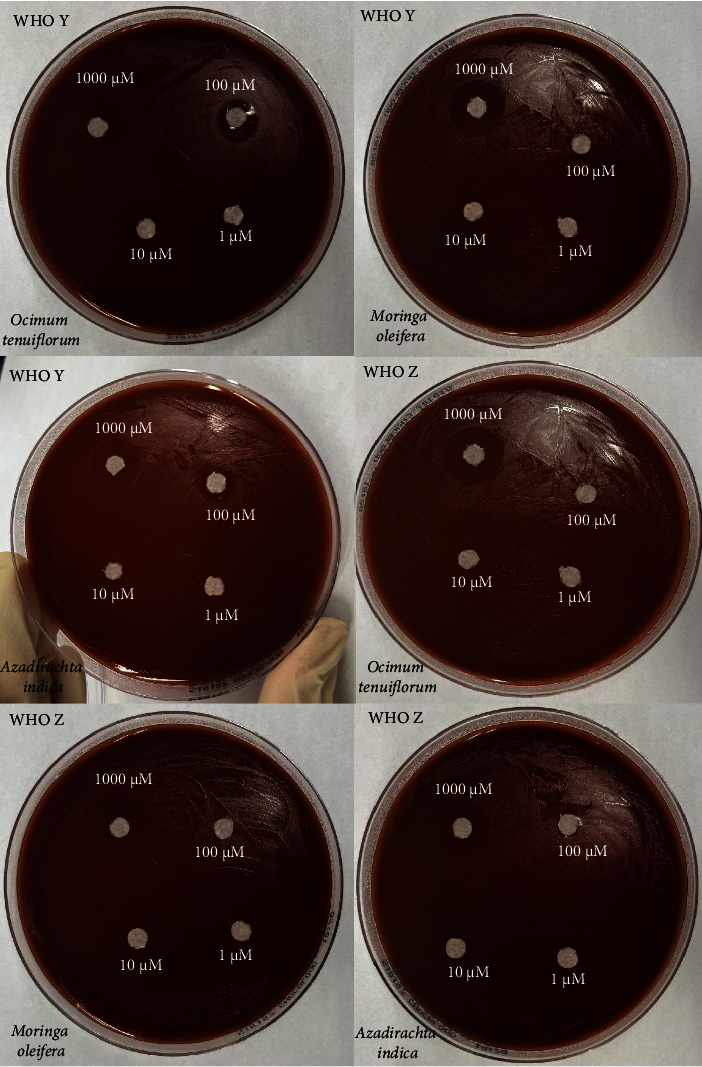
Antimicrobial activity of the nanoemulsions against the WHO control strains.

All six isolates had zones of inhibition for the 1000 *μ*M concentration for all three nanoemulsion based plant extracts. There were no zones of inhibition observed for 100 *μ*M, 10 *μ*M, and 1 *μ*M nanoemulsion concentrations for five of the isolates (Figures 3, 4, and 5). Isolate G176 had zones of inhibition at both 1000 *μ*M and 100 *μ*M concentrations for the nanoemulsions of *O. tenuiflorum* (Figure 3). WHO Y and WHO Z were used as a positive control (both resistant to Azithromycin and Ceftriaxone). Both the WHO strains had zones of inhibition appearing at the 1000 *μ*M concentration (Figure 6). For the WHO Y strain, zones of inhibition for both 1000 *μ*M and 100 *μ*M concentrations were observed for the nanoemulsions of *O. tenuiflorum* and *A. indica.* The radius of the zone of inhibition was recorded (Supplementary Table 1); however, since this was a novel study, there was no breakpoint data to compare with. The data described in this study may be used for future studies.

### 3.2. Haemolytic Activity of the Nanoemulsions

The haemolytic activity of the nanocomposite of the three plant leaf extracts (*O. tenuiflorum*, *M. oleifera*, and *A. indica*) was investigated using human erythrocytes at 1000 *μ*M. This concentration was selected since it produced the best results for inhibiting the growth of *N. gonorrhoeae*. According to the analysis, 0% haemolytic activity was observed, which suggests the nontoxic nature of the extracts (Table 1).

## 4. Discussion

The prevalence of antibiotic-resistant cases of *N. gonorrhoeae* surged by 124% between 2013 and 2019 [23]. According to the 2019 antimicrobial resistance threats report from the Centres for Disease Control and Prevention (CDC), *N. gonorrhoeae* has attained the status of a global urgent threat [24]. Furthermore, WHO classified cephalosporin-resistant and fluoroquinolone-resistant *N. gonorrhoeae* strains as high-priority antibiotic-resistant pathogens in 2020 [25]. Recent research has shown that the resistance rates for *N. gonorrhoeae* have reached alarming levels, with up to 21% resistance to ceftriaxone, 22% to cefixime, 60% to azithromycin, and a staggering 100% resistance to ciprofloxacin [6, 26]. Moreover, various studies, including those by Kueakulpattana et al. 2021 have documented sporadic instances of multi-drug resistant (MDR) and extensively drug-resistant (XDR) *N. gonorrhoeae* strains [27–29]. In response to this escalating global antibiotic resistance crisis, the recommended treatment regimen for noncomplicated gonococcal infections was revised in 2020 to include high-dose intramuscular ceftriaxone as the sole option. However, even this last resort is experiencing an increase in resistance rates [30].

Given the upward trajectory of antibiotic resistance observed in clinical *N. gonorrhoeae* strains and the extensive complications associated with asymptomatic gonorrhoea, the creation of a vaccine stands out as the most promising avenue for long-term protection against *N. gonorrhoeae.* Despite concerted efforts, the absence of a reliable vaccine or effective drug against highly drug-resistant *N. gonorrhoeae* remains a glaring reality. Consequently, it is imperative to continue striving for the development of novel and effective strategies to prevent and manage this pathogen. Due to the unsatisfying outcomes of newly developed antibiotics and the diminishing efficacy of repurposed older antibiotics, the pursuit of a new, potent antibiotic against *N. gonorrhoeae* becomes an unavoidable imperative. In response to increasing antibiotic resistance, researchers and healthcare professionals have been exploring alternative treatment options and strategies to combat gonorrhoea. Therefore, this study aimed to determine the antimicrobial activities of plant nanoemulsions from *O. tenuiflorum* (“holy basil”), *M. oleifera*, and *A. indica* plants against *N. gonorrhoeae* and to determine the toxicity of the plant nanoemulsions using human erythrocytes.

Many medicinal plants contain bioactive compounds with antimicrobial properties. These natural compounds have been used in traditional medicine for centuries and are often considered safer and less likely to lead to antibiotic resistance when compared to synthetic antibiotics. According to the WHO, traditional medicines derived from medicinal plants continue to provide benefits to 80% of the developing world's population [31–33]. Therefore, combining medicinal plant extracts with nanoemulsions can lead to synergistic effects. This study showed that at a concentration of 1000 *μ*M, the nanocomposites of *O. tenuiflorum*, *M. oleifera*, and *A. indica* inhibited the growth of *N. gonorrhoeae*. The haemolytic activity indicated that there was 0% haemolysis at 1000 *μ*M across all three plants. This showed that the nanoemulsion and plant mix may potentially be used as a safer alternative to treat gonorrhoea. Since this was the first study to investigate nanoemulsions combined with plant extracts against *N. gonorrhoeae*, our comparison with other studies is limited.

While the concept of using nanoemulsions combined with medicinal plant leaf extracts as an alternative treatment for *N. gonorrhoeae* is promising, it is important to note that further research is needed to determine the most effective plant extracts, the optimal nanoemulsion formulations, and the appropriate dosing regimens. Whilst the plant extracts used in this study (*O. tenuiflorum*, *M. oleifera*, and *A. indica*) are suggested to have antibacterial and anti-inflammatory properties and may show promising effects against *N. gonorrhoeae*, these plants, still require more comprehensive scientific research on their impact on *N. gonorrhoeae* and effect on the human body [14–18]. Although there are no human trials that have been published, there is experimental evidence that shows that holy basil may help in the treatment of various human bacterial infections including urinary tract infections, gonorrhoea, and Herpes [34–37]. Clinical trials and rigorous testing are necessary to ensure safety and efficacy before these alternative treatments can be widely adopted. However, the advantages of nanoemulsions can improve the solubility and stability of plant-derived compounds, while the plant extracts can provide potent antimicrobial activity. The synergy between plant extracts and nanoemulsions may enhance the effectiveness of the treatment against *N. gonorrhoeae*, potentially reducing the risk of resistance development. Another advantage to this approach is, that using nanoemulsions with plant extracts as an alternative treatment can help reduce the selective pressure for antibiotic resistance. By relying less on conventional antibiotics, we can slow down the evolution of antibiotic-resistant strains. Medicinal plant extracts are often considered safer and have fewer side effects when compared to some antibiotics. They may be better tolerated by patients, reducing the risk of adverse reactions. Medicinal plants are often readily available and sustainable, making them an attractive option for global healthcare, especially in regions where access to antibiotics is limited.

The current treatment of gonococcal infection according to the WHO guidelines for genital and anorectal infection is a dual therapy of ceftriaxone 250 mg intramuscular (IM) as a single dose plus azithromycin 1 g orally as a single dose or cefixime 400 mg orally as a single dose together with azithromycin 1 g orally as a single dose [4]. Therefore, keeping in line with the same treatment formulation, this study recommends that this formulation be administered as either an oral pill or intracellular injection as this STI is known to cause local and systemic infection [38, 39]. When the nanoemulsion with the plant extracts was tested for haemolysis, there was 0% haemolytic activity, therefore, indicating, and this approach as a possible safe alternative for the treatment of gonorrhoea. Therefore, administration of this nanocomposite in the form of an oral pill or intracellular injection may be safe for human prescription.

One limitation of this study lies in its relatively small number of isolates analysed. To enhance the robustness of future research, it is advisable to focus on a larger number of isolates, including a broader spectrum of gonococcal-resistant strains, to facilitate a more comprehensive analysis. Additionally, for a more thorough understanding, future investigations should explore a wider range of concentrations to determine the optimal treatment dosage. Another limitation of this study was that we did not investigate the nanoemulsion separately without the plant extracts. We investigated the nanoemulsion and plant extract as a composite. Therefore, we could not determine whether the nanoemulsion or plant extract alone had an antimicrobial effect. In the future, we will investigate separately which of the two components has a greater antimicrobial effect. An additional limitation of this study is that testing the toxicity only on erythrocytes is rather simple. We cannot say with certainty that these products are not toxic to the body only after these experiments. More complex studies would be needed to draw this conclusion when a larger study is conducted. Lastly, no controls were used (positive or negative control): the larger study can incorporate this. A notable strength of this study lies in utilising the WHO strains resistant to both azithromycin and ceftriaxone. Despite the broad resistance profiles of these strains, the nanoemulsion incorporating plant extracts effectively demonstrated the inhibition of growth of this pathogen in these gonococcal strains. This promising outcome suggests that the nanocomposite holds potential as an alternative treatment option for MDR gonococcal strains.

## 5. Conclusion

In conclusion, nanoemulsions paired with plant extracts (*O. tenuiflorum*, *M. oleifera*, and *A. indica*) offer a potential alternative to antibiotics for the treatment of *N*. *gonorrhoeae*. This approach harnesses the antimicrobial properties of natural compounds while leveraging nanoemulsion technology to enhance their delivery and effectiveness. However, ongoing research and development are essential to validate and refine this approach for clinical use.

## Figures and Tables

**Table 1:** Showing the haemolytic activity of plant extracts against human erythrocytes.

OD 540 nm of plants	*O. tenuiflorum*	*M. oleifera*	*A. indica*
*Concentration (μM)*			
1000	0.101	0.100	0.107
	0.100	0.112	0.105
	0.100	0.100	0.100

Average	0.1	0.1	0.1

Haemolysis (%)	0	0	0

The OD 540 reading for the PBS control was 0.1 and for the water control the reading was 0.291.

## Data Availability

The data used to support the findings of this research are included within the article.

## Supplementary Materials

Supplementary Materials Primary antimicrobial screening results using disk diffusion method (mm) for the nanoemulsion and plant extracts is included as a Supplementary Table 1.
