# Supplementary material for: Busting the Resistance: Antimicrobial Activity of Plant-Infused Nanoemulsions against Neisseria gonorrhoeae
Source: Int J Microbiol. 2024 Jul 30;2024:7084347. doi: 10.1155/2024/7084347 (PMC11303057; doi:10.1155/2024/7084347)
Supplement: Supplementary Materials — Primary antimicrobial screening results using disk diffusion method (mm) for the nanoemulsion and plant extracts is included as a Supplementary Table 1. [file 7084347.f1.docx]

**Supplementary**

**Supplementary Table 1:** Primary antimicrobial screening results using disk diffusion method (mm) for the nanoemulsion and plant extracts.

|  |  | Zone of inhibition (mm) | | | |
| --- | --- | --- | --- | --- | --- |
|  |  | Plant name | | |  |
| Isolates | Concentration (µM) | *Ocimum tenuiflorum* | *Moringa oleifera* | *Azadirachta indica* |  |
| G51 | 1000 | 8 | 8 | 10 |  |
|  | 100 | - | - | - |  |
|  | 10 | - | - | - |  |
|  | 1 | - | - | - |  |
| G136 | 1000 | 14 | 10 | 15 |  |
|  | 100 | - | - | - |  |
|  | 10 | - | - | - |  |
|  | 1 | - | - | - |  |
| G176 | 1000 | 10 | 8 | 10 |  |
|  | 100 | 5 | - | - |  |
|  | 10 | - | - | - |  |
|  | 1 | - | - | - |  |
| G180 | 1000 | 10 | 10 | 10 |  |
|  | 100 | - | - | - |  |
|  | 10 | - | - | - |  |
|  | 1 | - | - | - |  |
| G206 | 1000 | 15 | 14 | 10 |  |
|  | 100 | - | - | - |  |
|  | 10 | - | - | - |  |
|  | 1 | - | - | - |  |
| G247 | 1000 | 10 | 10 | 10 |  |
|  | 100 | - | - | - |  |
|  | 10 | - | - | - |  |
|  | 1 | - | - | - |  |
| Control  WHO Y | 1000 | 10 | 10 | 8 |  |
|  | 100 | 5 | - | 5 |  |
|  | 10 | - | - | - |  |
|  | 1 | - | - | - |  |
| Control  WHO Z | 1000 | 8 | 11 | 8 |  |
|  | 100 | - | - | - |  |
|  | 10 | - | - | - |  |
|  | 1 | - | - | - |  |
